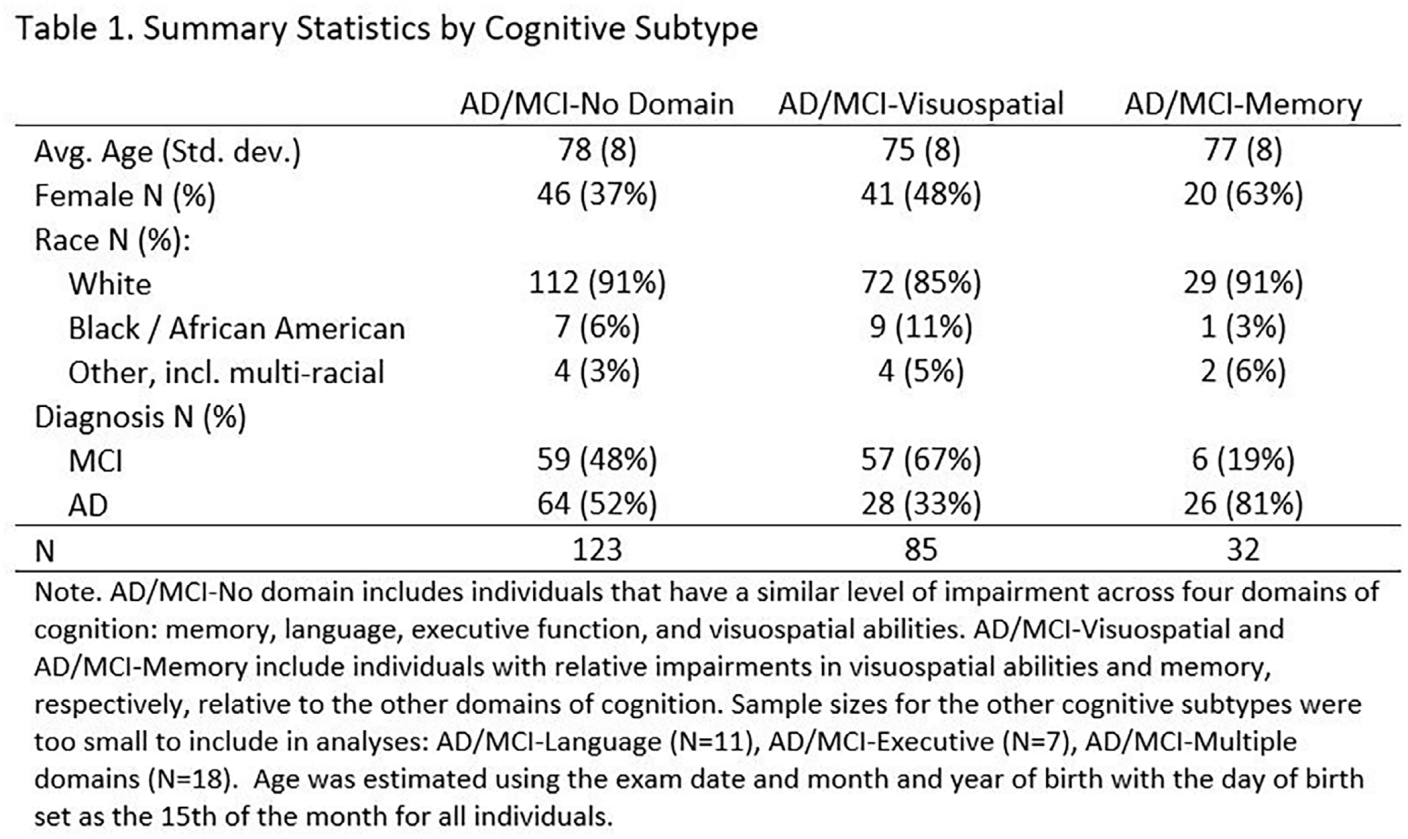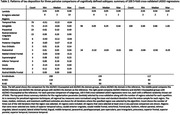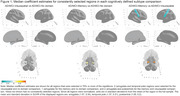# Patterns of Tau Deposition by Cognitive Subtype in the Alzheimer's Disease Neuroimaging Initiative

**DOI:** 10.1002/alz.093195

**Published:** 2025-01-09

**Authors:** Phoebe Scollard, Laura E. Gibbons, Seo‐Eun Choi, Michael L. Lee, Brandon S Klinedinst, Emily H. Trittschuh, Jesse Mez, Andrew J. Saykin, Connie Nakano, Elizabeth Sanders, Eardi Lila, Shannon L. Risacher, Elizabeth Mormino, Viktorija Smith, Mackenzie L. Carlson, Christina B. Young, Paul K. Crane, Shubhabrata Mukherjee

**Affiliations:** ^1^ University of Washington, School of Medicine, Seattle, WA USA; ^2^ University of Washington, Seattle, WA USA; ^3^ Department of Medicine, University of Washington, Seattle, WA USA; ^4^ Geriatric Research, Education, and Clinical Center, Veterans Affairs Puget Sound Health Care System, Seattle, WA USA; ^5^ Boston University, Boston, MA USA; ^6^ Boston University Alzheimer’s Disease Research Center, Boston, MA USA; ^7^ Indiana Alzheimer's Disease Research Center, Indianapolis, IN USA; ^8^ Indiana University School of Medicine, Indianapolis, IN USA; ^9^ Stanford University, Palo Alto, CA USA

## Abstract

**Background:**

Cognitive subtypes of Alzheimer’s Dementia (AD), defined by a relative impairment in a particular domain of cognition, have previously been shown to be associated with patterns of gray matter atrophy. Here we assessed the association of these subtypes with patterns of tau deposition measured in vivo using tau PET imaging in the Alzheimer’s Disease Neuroimaging Initiative (ADNI).

**Method:**

We included amyloid positive individuals with AD and Mild Cognitive Impairment (MCI). We selected the first diagnosis visit for AD and the most recent visit for MCI. Previously, AD individuals were categorized into AD‐Memory, AD‐Language, AD‐Executive, AD‐Visuospatial, AD‐Multiple domains, or AD‐No domain subtypes based on a relative cognitive impairment. These methods were extended to categorize MCI individuals. The AD/MCI‐Memory, AD/MCI‐Visuospatial, and AD/MCI‐No domain groups were large enough for our analyses. The tau PET scan closest to the subtyping visit was selected (median 49 days between scan and visit). Tau deposition for 35 brain regions (left and right sides averaged) were included as predictors. Separate five‐fold cross validated LASSO regressions were run for each of the three pairwise comparisons. Each model was repeated 100 times with different random fold selections to assess the stability of results.

**Result:**

We included 240 individuals (118 AD; 122 MCI) in our analyses (Table 1). There was some variation in the chosen models across repetitions with the AD/MCI‐Visuospatial versus AD/MCI‐No domain comparison varying the most (Table 2). We limit interpretation to those regions that appeared in ≥70% of repetitions. The amygdala was consistently selected in all pair‐wise comparisons. Higher tau deposition in this region was associated with a higher likelihood of being in AD/MCI‐Memory. Higher tau deposition in the postcentral region was associated with a lower likelihood of being in AD/MCI‐Memory compared to AD/MCI‐Visuospatial. Coefficients on consistently selected regions in the AD/MCI‐visuospatial versus AD/MCI‐No domain comparison were small. Figure 1 summarizes the top results from our analyses.

**Conclusion:**

We found heterogeneity in regional tau deposition among three cognitive subtypes. Future work will make use of additional cohorts with harmonized cognitive and imaging data. We plan to incorporate all cognitive subtypes and evaluate lateral asymmetry.